# Towards a Systems Immunology Approach to Unravel Responses to Cancer Immunotherapy

**DOI:** 10.3389/fimmu.2020.582744

**Published:** 2020-10-22

**Authors:** Laura Bracci, Alessandra Fragale, Lucia Gabriele, Federica Moschella

**Affiliations:** Tumor Immunology Unit, Department of Oncology and Molecular Medicine, Istituto Superiore di Sanità, Rome, Italy

**Keywords:** immunotherapy, chimeric antigen receptor T (CAR-T), immune-checkpoints, multi-parametric analysis, multi-omics, deep immunophenotyping

## Abstract

Immunotherapy, particularly immune checkpoint blockade and chimeric antigen receptor (CAR)-T cells, holds a great promise against cancer. These treatments have markedly improved survival in solid as well as in hematologic tumors previously considered incurable. However, durable responses occur in a fraction of patients, and existing biomarkers (*e.g.* PD-L1) have shown limited prediction power. This scenario highlights the need to dissect the complex interplay between immune and tumor cells to identify reliable biomarkers of response to be used for patients’ selection. In this context, systems immunology represents indeed the new frontier to address important clinical challenges in biomarker discovery. Through the integration of multiple layers of data obtained with several high-throughput approaches, systems immunology may give insights on the vast range of inter-individual differences and on the influences of genes and factors that cooperatively shape the individual immune response to a given treatment. In this Mini Review, we give an overview of the current high-throughput methodologies, including genomics, epigenomics, transcriptomics, metabolomics, proteomics, and multi-parametric phenotyping suitable for systems immunology as well as on the key steps of data integration and biological interpretation. Additionally, we review recent studies in which multi-omics technologies have been used to characterize mechanisms of response and to identify powerful biomarkers of response to checkpoint inhibitors, CAR-T cell therapy, dendritic cell-based and peptide-based cancer vaccines. We also highlight the need of favoring the collaboration of researchers with complementary expertise and of integrating multi-omics data into biological networks with the final goal of developing accurate markers of therapeutic response.

## Introduction

In the last few years, immune-based cancer therapies have been rediscovered as powerful clinical strategies against cancer. This breakthrough has begun with the discovery and clinical application of immune-checkpoint inhibitors (ICIs) that have changed radically the management of several types of once considered incurable cancers (1). The concept to target immune cells rather than cancer cells has achieved unexpected success especially with monoclonal antibodies against cytotoxic T-lymphocyte-associated antigen 4 (CTLA-4) and programmed cell death protein (PD-1) and its ligand PD-L1, improving enormously the survival rates of patients with metastatic melanoma, lung, renal, and urothelial cancers (2). In addition to ICIs, other immunotherapies such as peptide-based or dendritic cell (DC)-based vaccination and adoptive immunotherapy have been largely used, although clinical responses have been limited thus far (1, 3). Despite the beneficial effects of immunotherapies, immune-related adverse events are often observed and only a minority of patients display long-term responses (4, 5). Several variables determine the efficacy of immunotherapies and in particular, of ICIs. Among the others, one of the most important is the heterogeneity of the host immune response both at the tumor level and in the circulation (6). The nature and function of peripheral and tumor-infiltrating immune cell populations as well as tumor immune signatures and neo-antigen burden dynamically shape the immune contexture driving therapeutic responses (7). However, none of the above-mentioned parameters uniquely associates to immunotherapy responsiveness (8). Therefore, the development of models predicting therapy efficacy by identifying and correlating specific immune elements and functions at the time of diagnosis is central for selecting patients that would benefit from immunotherapeutic treatments. Systems immunology has the objective to generate reliable models predicting therapeutic responses and outcomes by integration of multi-layers immune analyses and advanced bioinformatics approaches (9). In the present review, we will provide an overview of the key technological approaches exploited by systems immunology analysis and will provide examples of application in the urgent search for reliable markers to select cancer patients for personalized immunotherapy approaches (10).

## Overview of Current High-Throughput Technologies for Systems Immunology

**Figure 1:**
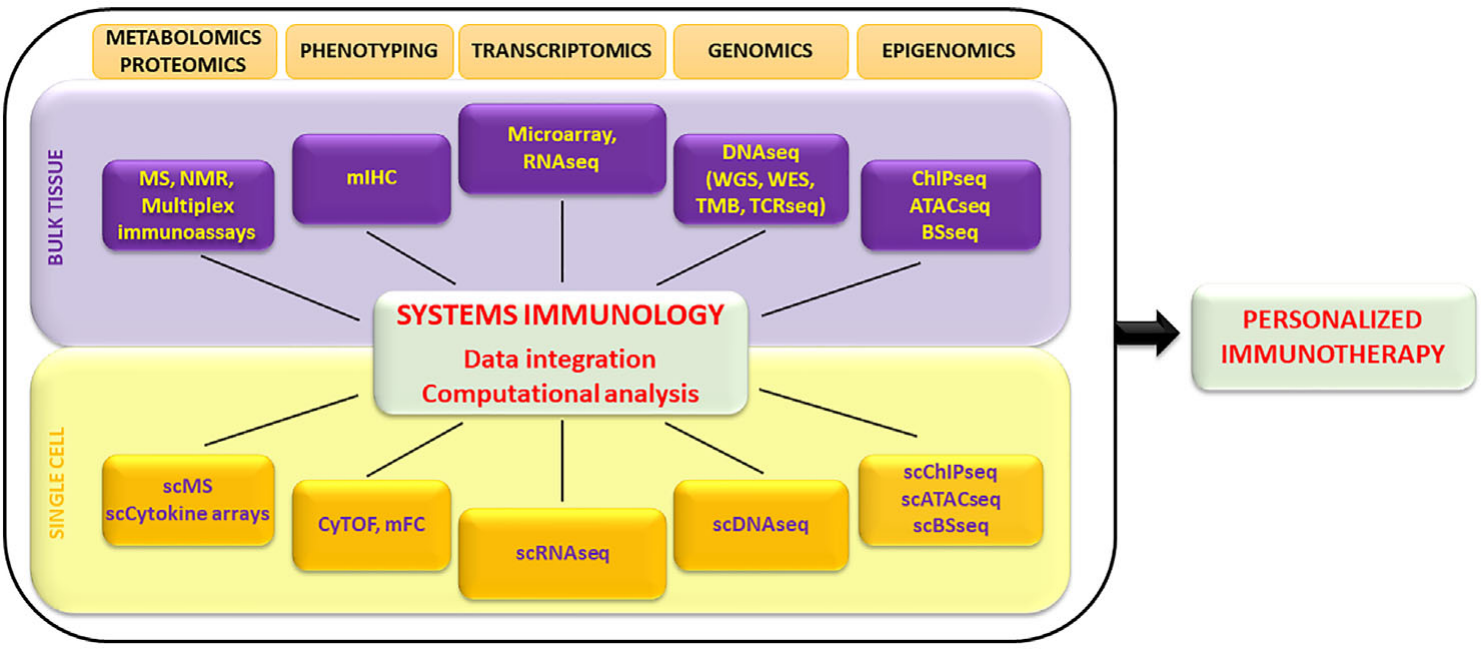
Overview of high-throughput technologies exploited by systems immunology to characterize the immune system at bulk tissue and single-cell levels. Large datasets are integrated by computational analysis and algorithms and allow the identification of biomarkers exploitable to design personalized immunotherapy treatments. MS, mass spectometry; NMR, nuclear magnetic resonance; mIHC, multiplex immunohistochemistry; RNAseq, RNA sequencing; DNAseq, DNA sequencing; WGS, whole genome sequencing; WES, whole exome sequencing; TMB, tumor mutational burden; TCRseq, TCR sequencing; ChIP-seq, chromatin immunoprecipitation sequencing; ATAC-seq, assay for transposase-accessible chromatin using sequencing; BS-seq, bisulfite sequencing; CyTOF, cytometry by time of flight; mFC, multiparametric flow cytometry.

Several high-throughput technologies are used to characterize the immune status of cancer patients either before or throughout immunotherapy treatment (**Figure 1**). In the past years, gene expression profiling by microarray was extensively used to evaluate the transcriptome of tissues in numerous biomedical investigations (11). It employs a collection of DNA spots attached to a solid surface. One of the limitations of microarray is the large amount of input RNA required, and it is therefore usually applied to whole blood or to heterogeneous tissue samples. More recently, RNA sequencing (RNA-seq) has completely revolutionized transcriptomic analysis. RNA-seq protocols in general involve the following steps: isolation of RNA, reverse transcription (RT), amplification, library generation, and sequencing (12). RNA-seq has several advantages over microarray technology: i) high coverage and sensitivity (detecting low-abundance transcripts), ii) detection of small RNAs, iii) low background noise and batch effects, iv) low RNA input (12). However, bulk-based profiling performed by averaging results from thousands of cells of distinct types represents a problem for data analysis and interpretation. The advent of single-cell-RNAseq (scRNA-seq) overcame this challenge by quantifying molecular features at the single-cell resolution (13). Individual cells are encapsulated in droplets in a microfluidic device, where the RT reaction takes place. Each droplet carries a “barcode” that uniquely labels the cDNAs derived from a single cell. The ability to read and annotate transcriptomes at single-cell resolution has been coupled with the development of computational methodologies for data analysis and processing that present several challenges (14). Recently, the SIDEseq has been introduced as new measure to evaluate pairwise similarities between cells using scRNA seq data (15). The SIDEseq identifies the lists of putative differentially expressed genes (DEGs) between each pair of cells and uses the consistency between the two lists of DEGs to define their similarity. Through the analysis of simulated and real datasets with varying degrees of complexity, the SIDEseq allows the identification of thin but meaningful differences between small cell subpopulations.

A new technology termed “spatial transcriptomics” allows visualization and quantitative analysis of the transcriptome with spatial resolution in individual tissue sections. It uses primers of cDNA spatially barcoded for full-transcriptome capture on tissue sections giving a comprehensive 2D or 3D visualization of all mRNAs in tissue sections (16). This method is easy to perform and can be applied alone or in combination with single-cell technologies.

Epigenomics together with transcriptomics enlarge the characterization of the heterogeneity, plasticity, and functional diversity of the immune system as transcriptional regulation is thought to be the proximal effect of epigenomic modifications. Chromatin immunoprecipitation (ChIP-seq) reveals DNA binding sites of specific transcription factors or histone modifications by IP with specific antibodies (Abs). Recently, a high-throughput droplet microfluidics platform to profile chromatin landscapes at single-cell resolution has been described (17). Assay for Transposition of Accessible Chromatin (ATAC-seq) is an alternative to DNase-seq that uses an engineered Tn5 transposase to cleave DNA and to integrate primer DNA sequences into the cleaved genomic DNA. Notably, ATAC-seq has opened the door to the study of tumor-infiltrating lymphocytes (TILs) from the epigenetic perspective (18). Improvements of ATAC-seq have been reported (19).

Chromatin remodeling can be revealed by the analysis of methylome. Pyrosequencing and direct sequencing have been the most widely used methods for analysis of promoter region or CpG islands. The limitations of these techniques include low quantitative accuracy, short read length, and low sample throughput. Emerging next generation sequencing (NGS) platforms allow for massive analysis of the methylation status of almost every CpG site and construction of DNA methylation’s genomic maps at a single base resolution. Modern techniques for DNA methylation mapping use the same software as those that were developed for genetic and genomic analyses (20).

Many reports have shown that the tumor mutational burden (TMB) correlates with the response to ICIs (21). Non-synonymous mutations, in fact, can produce novel tumor-specific antigens (neoantigens) recognized by the host immune system. Although whole exome sequencing (WES) is the gold standard technique, given the global measurement potential, its clinical use is hampered by high cost. Panel sequencing, with various approaches to extrapolate the global TMB from the narrow sequencing, is more used in clinical application and includes several oncopanels, such as Trusight170, Oncomine Tumor Mutation Load Assay, MSK-IMPACT and FoundationOne (22). Possible neoantigens are then predicted *in silico* according to their affinity to the MHC class I alleles (23).

The adaptive immune response generates a large repertoire of T and B cells with different T-cell receptors (TCRs) and B-cell receptors/immunoglobulin (BCRs/Ig). The repertoire changes in response to different antigens (24), diseases and therapies (25). Conventional methods include: i) spectratyping, which analyzes the variation in the lengths of RT-PCR products generated from the third complementarity-determining region (CDR3) region in TCR V*β* family (26) and BCR/Ig heavy chain, ii) flow cytometry, and iii) immunohistochemistry (IHC). To overcome the limited sensitivity/accuracy of these methods, high-throughput NGS has been developed to profile TCR and BCR/Ig repertoires at the single-cell level. TCR-seq (27) and BCR/Ig-seq (28) is a three-step process: i) PCR amplification of V–D–J (for TCR*β*, TCR*δ*, and IgH) or V–J (for TCR*α*, TCR*γ*, and IgL) gene segments, ii) massive parallel sequencing of the PCR amplicons, and iii) alignment of the reads by bioinformatics.

It has been recognized that the type and density of TILs is an important prognostic parameter in numerous cancer types (29). Compared to traditional single color IHC, multiplexed IHC (mIHC**)** enables the contemporary evaluation of multiple parameters from a single tissue section (30). Of note, a seven-color multispectral IHC of tumors from patients with melanoma could select patients for successful TIL generation for adoptive immunotherapy protocols (31).

Multi-parametric flow cytometry (mFC) is the gold standard technique for enumerating and identifying cells within heterogeneous biological samples. It offers the opportunity to comprehensively characterize the immunologic state of patients on a single-cell basis by using combinations of fluorescently labelled monoclonal Abs specific for target molecules. mFC offers the possibility to differentiate between up to 30 parameters, but as opposed to IHC, it does not provide information about the spatial distribution of a given subset in the analyzed sample. The advent of mass cytometry (cytometry by time-of-flight, CyTOF) has revolutionized human immune cell profiling. It allows the simultaneous measurement of >40 markers on a single-cell basis. This is achieved by using antibodies coupled to rare metal isotopes providing a unique mass tag for each marker, which is detected by time-of-ﬂight mass spectroscopy (32). The number of markers and the absence of relevant spectral overlap are a major advantage over mFC. CyTOF is particularly valuable when analyzing samples with a limited number of cells such as pediatric patients or tumor biopsies. In a recent report, Gadalla and colleagues demonstrated that using a 40+ parameter panel on peripheral blood mononuclear cell (PBMC) and tumor tissue samples, CyTOF is as effective as mFC for the identiﬁcation of diverse cell subsets and their subsequent phenotyping (33).

Given the key role of metabolites in modulating T cell function (34) and in affecting the success of immunotherapies (35), a comprehensive analysis of metabolites can effectively complement molecular and phenotyping studies. Metabolomics studies are based on two major technologies, mass spectrometry (MS) and nuclear magnetic resonance (NMR) (36). MS can perform both targeted and untargeted analyses. Targeted analyses follow hundreds of known molecules and quantify key known compounds. Untargeted metabolomics profile many thousands of features globally and can discover novel biomarkers found in specific conditions. Recent advancement in NMR technology has improved its sensitivity and the availability of databases has facilitated the identification of molecules (37).

Proteome profiling allows the contemporary quantification of hundreds of proteins in cell extracts or body fluids. The latter, obtainable through noninvasive procedures, are of particular relevance for biomarker discovery. MS has been extensively used for comprehensive proteome profiling (38). Alternative methods, including high-throughput multiplex immunoassays (Luminex, Bio-Plex) and single cell arrays for secreted cytokines (39), focus on the detection of key factors regulating immune response and intercellular communication, such as cytokines, chemokines, and inflammatory mediators.

## Integration of Large Datasets and Biological Interpretation

All the above described high-throughput technological approaches create an enormous amount of data that needs to be computationally analyzed and examined with statistical algorithms for extracting biologically relevant information. The first step in this process is data integration, which involves reformatting the results from multiple assays so that the data can be analyzed as an integrated whole. Data are further analyzed with sophisticated mathematical and computational algorithms to infer biological relationships and to correlate the data with the clinical outcome. By selection of variables of interest potentially predicting patient outcome, these steps are crucial to generate a scientific hypothesis that needs to be tested and validated (40). Various bioinformatics data analysis tools have been developed to organize data from multiple high-throughput assays. However, given the complexity of the immune system and the non-linear correlation of interconnected immune signals, each approach presents advantages and disadvantages. As an example, the analysis of transcriptome data obtained from RNA-seq or microarray experiments are generally carried out according to a bioinformatics workflow including differential gene expression analysis, functional or pathway enrichment, gene set enrichment analysis. This analytic pipeline leads to data visualization of gene expression patterns to be correlated to immune functions (41). Relevant to the analysis of immune transcriptome data are the deconvolution methods that allow quantifying the relative fractions of the immune cell types of interest (42). However, as deconvolution algorithms rely on the assumption that the expression of a gene in a mixture is the result of a linear combination of the expression of that gene in the different cell types, advanced and efficient algorithms remain to be developed to capture the nonlinear cell-cell correlations. This concept supports the increased relevance of high-throughput assays and data collection at single-cell resolution. A recent review highlighted examples of data-driven systems modeling that characterize, map, or connect components of the immune system, with application in cancer immunotherapy (43). In the next sections, we will provide examples of the application of one or more high-throughput multi-parametric technologies for the identification of predictors of response to immunotherapy.

## Integrative, Multi-omics Approaches to Unravel Responses to ICIs and Identify Predictive Biomarkers

The comprehensive analysis of patients’ immune status by systems immunology uniquely offers the possibility to understand the complex interplay between tumor and host cells in the context of successful or ineffective responses to ICIs and allows the identification of biomarkers for personalized immunotherapy treatments. Several recent studies have reported unbiased high-throughput analyses in the attempt to identify immune correlates of response to ICIs. In a small cohort of non-small cell lung cancer (NSCLC) patients treated with anti-PD-1 Abs, expression signatures of immune-related genes were correlated with durable clinical benefit in 9 out of 21 patients (44). Of note, up-regulation of macrophage 1 and down-regulation of peripheral T cell gene signatures showed the best performance for discriminating between durable and non-durable clinical responses. In the same tumor histotype, the response to anti-PD-1 Ab was also shown to correlate with the molecular smoking signature, high candidate neoantigen burden and DNA repair pathway mutations (45). In small cell lung cancer high TMB was associated with improved objective response, durable clinical benefit, and better progression-free survival following PD-1 and CTLA-4 blockade (46). Likewise, TMB, the candidate neoantigen load and the expression of cytolytic markers in the TME were significantly associated with clinical benefit in melanoma patients in response to CTLA-4 Ab (47, 48). More recently, TMB and efficient neoantigen presentation analyzed by NGS in a cohort of 83 patients with 20 different solid malignancies revealed predictors of response to ICIs (49). In another study, a deep machine-learning model integrating TMB, microsatellite instability, and somatic copy-number alterations was used to subclassify 8,646 tumors from 29 tumor types into four distinct genomic clusters (50). Each cluster was associated with a unique immune landscape inferred by deconvolution of RNA-seq TGCA datasets, highlighting the complex relationship between the tumor genomic landscape and host immunity. Most importantly, applying this model to tumors from metastatic melanoma patients treated with ICIs demonstrated that different genomic clusters were associated with distinct clinical responses to ICI treatment. Along these lines, the integration of mFC, gene expression, and mIHC revealed in lung cancer specimens the presence of a myeloid-rich subgroup enriched in neutrophils, correlating with the absence of intratumor T cells, and identified tumor CD8^+^/neutrophil ratios as predictors of ICI treatment responsiveness (51). In another study, among the 36 parameters analyzed across 21 cancer types, the estimated CD8^+^ T-cell abundance in TME was the most predictive of the response to anti-PD-1/PD-L1 therapy, followed by the TMB and by the fraction of samples with high PD-1 gene expression. Of note, the combination of these parameters highly correlated with response to anti-PD-1/PD-L1 treatment (52). In melanoma patients treated with anti-PD-1 Abs, immuno-profiling of PBMC by high-dimensional single-cell CyTOF was used to investigate circulating immune correlates of response. Indeed, the authors observed a more prominent increase in monocyte (CD14^+^CD16^−^CD33^+^HLA-DR^+^) frequency in responders as compared to non-responders, thus indicating that blood-based biomarkers can also be relevant in clinical practice (53).

## Systems Immunology and Biomarker Discovery in Patients Treated With Other Immunotherapies

Among the most promising immunotherapies, chimeric antigen receptor (CAR-T) cell therapy has seen exceptional success in several hematologic malignancies. Yet, challenges in translation to solid tumors still exist. The effectiveness of adoptive immunotherapy with CAR-T cells depends on a complex interplay of tumor, immune and stromal cells, which systems immunology approaches may help to fully elucidate. In a recent study, TCR-seq, integration site analysis, and scRNA-seq were used to profile anti-CD19 CAR-T cells before and after infusion, revealing that clonal diversity declines following infusion and that expanding clones have higher expression of proliferation and cytotoxicity genes (54). By analyzing the transcriptional and cytokine signatures (by means of scRNA-seq and single-cell multiplex cytokine secretion assay), together with live cell imaging of cytotoxic activity, Xhangolli et al. demonstrated that anti-CD19 CAR-T cells display a highly mixed Th1/Th2 function upon antigen-specific stimulation (55). Lymphodepleting chemotherapy is routinely administered prior to CAR-T cell infusion, and has been associated with improved *in vivo* cell expansion and persistence (56). We used microarray analysis and cytokine protein profiling to unravel the complex systemic effect of preconditioning chemotherapy, revealing the importance of a type I interferon signature and of distinct cytokine profiles in the response to adoptive immunotherapy (57–59). Remarkably, the cytokine profile induced by preconditioning chemotherapy was shown to correlate with progression-free survival in patients treated with anti-CD19 CAR-T cells (60). An integrated systems immunology approach in the context of CAR-T cell therapy evaluating at the same time the characteristics of the cell product and the chemotherapy-induced immune profiles is still lacking, and it is expected to be crucial to improve the therapeutic efficacy of CAR-T cell therapies.

Although systems immunology studies are less common in the context of immunotherapies other than ICIs and CAR-T, several recent omics studies have contributed to elucidate mechanisms underlying successful DC-based and peptide-based anticancer vaccination. In malignant pleural mesothelioma patients undergoing DC-based vaccination, changes in TCRβ repertoire of circulating lymphocytes revealed by NGS, significantly correlated with patient survival (61). In melanoma patients treated with adenovirus-transduced DC and high-dose systemic interferon-alpha-2b (IFN-*α*2b), a detailed phenotypic and functional analysis of blood NK cells was carried out by mFC, multiplex gene expression analysis and serum content analysis. The results demonstrated that CD56^dim^CD16^−^NK cells are a unique non-cytolytic subset that may positively impact clinical outcome (62). In a randomized clinical trial in which resected stage III–IV melanoma patients were treated with peptide-based vaccination and IFN-*α*2b, with or without dacarbazine preconditioning, we have used mFC to reveal parameters correlating with relapse-free survival. Our treatments induced an increase of polyfunctionality and of IL-2 production by vaccine-specific CD8^+^ T cells and an expansion/activation of NK cells (CD56^dim^CD16^-^CD107a^+^) only in relapse-free patients (63). Altogether, these results show that high-throughput multi-omics technologies are effective to evaluate therapeutic effects and may be used to guide therapeutic interventions.

## Conclusions

Systems immunology provides unprecedented opportunities for biomarker discovery stemming from the integration and statistical analysis of large datasets generated by high-throughput analysis of biological samples either at single cell or at bulk tissue level. Notwithstanding the remarkable present achievements of high-throughput technologies and of *in silico* analysis, their full potential to revolutionize immunotherapy has yet to be fully realized. In fact, the vast majority of recent immunotherapeutic studies still rely on the use of a single omic approach at a time. Indeed, integration of large datasets characterized by inherent format differences generated by multiple omic platforms is the main bottleneck of systems immunology. Meaningful biological interpretation of large and heterogeneous datasets requires a constant evolution of databases and data analysis tools as well as the collaboration of researchers with complementary expertise to carry out multidisciplinary analysis capable of integrating the emerging data into clinically applicable predictive algorithms. In addition, since measurements typically involve multiple platforms across multiple laboratories, standardization and harmonization efforts are needed to allow comparison of results and maximize the clinical translation of results. It’s now emerging that the density, spatial distribution, and functionality of tumor-infiltrating immune cells as well as the presence of other non-immune components of the TME (endothelial cells, cancer-associated fibroblasts, *etc*) are important predictors of response to immunotherapy. The future of research for biomarkers of response to immunotherapy is therefore expected to rely on the analysis of the TME as a whole, rather than focusing on the analysis of single components. In this respect, phenotypic and functional analysis of tumors by mFC or CyTOF may be complemented by high-throughput spatial transcriptomics (16) to gain insights on the relative abundance and spatial distribution of cell subtypes in the TME. A promising perspective is also represented by the application of NGS or deep immunophenotyping technologies to liquid biopsies to identify circulating biomarkers (cancer-derived DNA, circulating tumor cells, exosomes or immune cells) of great potential clinical utility due to the non-invasiveness and repeatability of their measurement. Overall, we surmise that the continuous implementation of technologies supporting the advancement of systems immunology represents an important frontier for understanding human immunity and foresee its enormous potential to revolutionize cancer treatment in the near future.

## Author Contributions

LB conceptualized the manuscript. LB, AF, and FM led the review process and wrote the original draft. LG made substantial contributions to discussions of the content and revised the manuscript. AF contributed to image conceptualization and design. All authors contributed to the article and approved the submitted version.

## Funding

We acknowledge the Italian Ministry of Health (Ricerca Corrente).

## Conflict of Interest

The authors declare that the research was conducted in the absence of any commercial or financial relationships that could be construed as a potential conflict of interest.
